# Serial cultures in invert emulsion and monophase systems for microbial community shaping and propagation

**DOI:** 10.1186/s12934-024-02322-3

**Published:** 2024-02-14

**Authors:** Alexis Dijamentiuk, Cécile Mangavel, Chloé Gapp, Annelore Elfassy, Anne-Marie Revol-Junelles, Frédéric Borges

**Affiliations:** Laboratoire d’Ingénierie des Biomolécules (LIBio), Université de Lorraine, Nancy, France

**Keywords:** Serial passaging, Microbial community, Coculture systems, Microbiome engineering, Community structure, Enrichment culture, Raw milk

## Abstract

**Background:**

Microbial communities harbor important biotechnological potential in diverse domains, however, the engineering and propagation of such communities still face both knowledge and know-how gaps. More specifically, culturing tools are needed to propagate and shape microbial communities, to obtain desired properties, and to exploit them. Previous work suggested that micro-confinement and segregation of microorganisms using invert (water-in-oil, w/o) emulsion broth can shape communities during propagation, by alleviating biotic interactions and inducing physiological changes in cultured bacteria. The present work aimed at evaluating invert emulsion and simple broth monophasic cultures for the propagation and shaping of bacterial communities derived from raw milk in a serial propagation design.

**Results:**

The monophasic setup resulted in stable community structures during serial propagation, whereas the invert emulsion system resulted in only transiently stable structures. In addition, different communities with different taxonomic compositions could be obtained from a single inoculum. Furthermore, the implementation of invert emulsion systems has allowed for the enrichment of less abundant microorganisms and consequently facilitated their isolation on culture agar plates.

**Conclusions:**

The monophasic system enables communities to be propagated in a stable manner, whereas the invert emulsion system allowed for the isolation of less abundant microorganisms and the generation of diverse taxonomic compositions from a single inoculum.

**Supplementary Information:**

The online version contains supplementary material available at 10.1186/s12934-024-02322-3.

## Background

Microbiomes, defined as microbial communities characterizing a specific habitat as well as their “theatre of activity”, harbor critical roles in the biogeochemical cycling of natural resources [1–4]. They also represent a large reservoir of biological and functional diversity, the understanding of which is increasingly recognized as crucial to the development of a global, sustainable bioeconomy [5]. Culture-independent multi-omics approaches have revealed the immense diversity of the bacterial repertoire of microbiomes and shed light on their functioning. These advances have fueled strong interest in microbiome engineering in order to improve the biotechnological potential of microbial communities. It is nowadays well recognized that microbial communities represent an opportunity for applications covering numerous domains such as biocontrol of crop cultures [6], anaerobic digestion [7] or biopreservation of food matrices [8]. Engineering microbiomes requires rational approaches in order to obtain the desired properties. A conceptual framework has been proposed to achieve this goal by defining an integrated process combining *bottom-up* (rational assembly of microbial communities) and *top-down* (act on environmental variables to force a microbiome to perform a desired process or function) approaches [9].

These new perspectives emphasize the importance of microbial cultures and underline the need for innovative tools and culture processes to build and manipulate microbial communities. Top-down engineering can rely on *ex-situ* cultivation of naturally occurring communities [10–12]. This approach involves the manipulation of environmental pressures in order to modulate the structure and functionality of microbial communities. To this end, it is possible to tune the culture process parameters such as temperature, nutrient composition of the substrate as well as incubation regime [13]. In a synthetic biology perspective, top-down engineering can also involve the use of co-culture systems in which microbial populations are allowed to grow with some degree of proximity or contact between them, modulating the intensity of biotic interactions [14]. As an example, it is possible to immobilize cells in bioreactors for continuous lactic acid fermentation in fresh cheese manufacturing [15]. The system consisted in inoculating a bioreactor containing UHT milk inoculated with four strains of lactic acid bacteria, separately or encapsulated together in κ-carrageenan beads and locust bean gum gel so as to decrease bacterial interactions. Cell immobilization improved both lactic acid and cell yields over a homogeneous mixed culture for several weeks [16]. Alternatively, water-in-oil emulsion (invert emulsion) co-culture systems have been used for stochastic segregation of bacteria in nurturing droplets, alleviating interaction between microorganisms [14, 17, 18]. This process, applied at the population level within the species *Lactococcus lactis*, allowed the gradual enrichment of slow-growing mutants, whereas in monophasic culture they were rapidly excluded [17]. However, when used at the scale of a synthetic consortium composed of six different bacterial species, a similar invert emulsion system led to a distortion of the initial structure during a single propagation step, although it also allowed a better maintenance of diversity compared to a monophasic culture [19]. Serial propagation is usually necessary in top-down community engineering, and it has previously been shown that serial propagation in monophasic setups tends to stabilize community structures [10, 20–23]. In contrast, the effect of serial propagation in invert emulsion on the structure of complex consortia is not known.

The aim of this work was to investigate the effect of invert emulsion culture system, compared to monophasic culture, on the structural dynamics of bacterial communities during serial propagation. To address this issue, three different raw milk-derived microbiota were serially propagated using both culture systems and the resulting community structures were investigated by metabarcoding.

## Materials and methods

### Invert emulsion culture system

Cultures in invert emulsions were prepared under sterile conditions as previously described [19] by dropwise addition of 3 mL inoculated medium to a mix of 11.625 g (77.5% w/w) refined sunflower oil (Système U, Rungis, France) and 375 mg (2.5% w/w) polyglycerol polyricinoleate (PGPR) (Paalsgaard A/S; Julsminde, Denmark) in a 40 mL, 30 mm diameter vial (CEB, Angers, France). The emulsification was carried out with stirring using a magnetic stirrer (IKA-Werke, GmbH & Co. KG, Staufen, Germany) and a polygonal stir bar with pivot ring (L: 20 mm, Ø 6 mm) at 400 rpm and 20 °C during 5 min. The invert emulsions were then transferred into 15 mL CELLSTAR® centrifugation tubes (Greiner Bio-One International GmbH, Kremsmünster, Austria) and incubated at 30 °C for 24 h. To avoid either sedimentation or excessive shearing of the samples, cultures were held on a Stuart SB-2 tube rotator (Cole-Parmer LLC, Vernon Hills, USA) with a 45° tilt, continuously rotating at 20 rpm. The breaking of invert emulsions was carried out in several steps [19]: 1.5 mL samples were centrifuged at 5,000 g for 10 min. The oil phase was then removed and 300 µL of 1*H*, 1*H*, 2*H*, 2*H*-perfluoro-1-octanol (Alfa Aesar, Ward Hill, MA, USA) were added. Phase separation occurred after 15 min of gentle shaking followed by settling.

### Serial propagation of microbial communities derived from raw milk

Three raw milk samples stored at -30 °C, named LC, L5 and L52 were used in this study. Inoculations were performed by diluting thawed aliquots of milk in fresh Trypticase Soy Broth (TSB) (BioMérieux, Marcy-l’Etoile, France) supplemented with 6 g.L^− 1^ of Bacto™ yeast extract (YE) at a ratio of 1:100 so that the initial population density did not exceed 10^7^ CFU.mL^− 1^ in invert emulsion systems, as previously described [19]. Serial propagation consisted of 10 growth cycles, starting with the suspension of 300 µL of raw milk in 30 mL of TSBYE. Each growth cycle consisted of four steps. (1) The inoculated broth was homogenized and divided in two parts: 15 mL were used for monophasic culture, and 3 mL were used for the production of 15 mL invert emulsion culture. (2) Both cultures were transferred into 15 mL centrifugation tubes, then placed onto the tube rotator and incubated during 24 h at 30 °C. (3) After incubation, 300 µL of grown and homogenized monophasic culture or aqueous phase of broken emulsified culture were suspended in 30 mL fresh broth. After initial inoculation and each culture step, cultures were enumerated and used for DNA extraction and subsequent metabarcoding analysis.

### Bacterial enumeration

Aliquots of raw milk were thawed, homogenized, and enumerated before use for cultivation experiments. Enumeration consisted of serially diluting bacterial suspensions in tryptone-salt buffer (Biokar Diagnostics, Paris, France) amended with 2% Tween® 80 (Merck KGaA, Darmstadt, Germany) and plating onto TSBYE containing 15 g.L^− 1^ agar (TSAYE). Plates were then incubated at 30 °C for 72 h before reading. Given the proportion of aqueous medium in the invert emulsion (1:5), raw cell numbers in emulsified cultures were corrected by a factor of 5.

### DNA extraction

DNA extraction was performed using the NucleoSpin Food® kit (Macherey-Nagel, Düren, Germany) with modifications [19]. It consisted of centrifuging 1.5 mL of bacterial suspension for 10 min at 10,000 g, then removing the supernatant. Then, the pellet was suspended in 550 µL of 65 °C pre-warmed lysis buffer from the kit and 10 µL proteinase K was added (10 mg.mL^− 1^), as well as 10 µL of 20 mg.mL^− 1^ RNase and approximately 200 to 300 mg of sterile, UV-treated, 150 to 212 μm diameter glass beads. After incubation at 65 °C for 3 h, the reaction mix was shaken at 3,200 rpm on a Vortex Genie 2 horizontal agitator (Scientific Industries, New York, USA) for 1 h at room temperature, then centrifuged at 10,000 g for 10 min. DNA purification from the supernatant was then conducted following the instructions of the NucleoSpin Food® kit.

### Sequencing and downstream analysis

PCR, amplicon normalization, paired-end sequencing, sequence merging as well as quality control were outsourced to Eurofins Genomics Europe GmbH (Konstanz, Germany) following the InView-Microbiome Profiling 3.0 procedure. Briefly, the V3-V4 hypervariable region of the bacterial 16S rRNA was targeted by the forward 357F (5’-TACGGGAGGCAGCAG-3’, [24]) and reverse 800R (5’-CCAGGGTATCTAATCC-3’, [25]) primers and amplified using myTaqTM HS DNA Polymerase (Meridian Bioscience Inc., Cincinnati, Ohio, USA). Illumina (MiSeq) sequencing was conducted according to Kozich et al. [26] and produced 2 × 300 bp reads with a minimal depth of 60,000 reads. Paired-end reads were merged using the software FLASH version 2.2.00 [27] with a minimum overlap size of 10 bp. Merged sequences were approximately 425 bp in length. Further processing of sequences was done with the FROGS tools [28] on the Galaxy Migale plateform (Galaxy version 3.2.3). The denoising step consisted of removing reads that did not match the expected length (i.e. between 350 and 450 bp) and the ones containing ambiguous bases (N). After dereplication, the clustering step was performed with the fastidious option in SWARM, a single-linkage clustering algorithm using a local clustering threshold (aggregation distance of 1 in this work) [29]. After the removal of chimeras, OTUs with an abundance as proportion inferior to 5.10^− 5^ were removed as recommended by Bokulich et al. [30]. Taxonomic affiliation was then performed by BLAST against the 16_EZBioCloud_52018 database [31]. A post-processing step aggregated OTUs sharing the same taxonomy with at least 99% identity and 99% alignment coverage. The abundance table was then imported into R version 4.1.2 [32] for further analysis. Taxonomies have been modified according to recent reclassification and renaming [33, 34].

### Analysis of community structure

Alpha and beta diversity analyses were conducted using tools from the vegan R package version 2.6-4 [35]. Alpha diversity analysis consisted in calculating the Shannon index. The mean Shannon indices of the communities from the L5, L52, and LC milk samples were calculated for each growth step. The measurement of the dispersion of each series (milk and culture system) between steps 2 and 10 was normalized by calculating the coefficient of variation (CV, i.e. the ratio of the standard deviation to the mean). The values obtained were used to calculate the mean dispersion and the standard error of the mean for each cultivation modality. In the beta diversity analysis, rarefied Bray-Curtis dissimilarities were obtained using the *avgdist* function. Briefly, the raw abundance OTU matrix was randomly subsampled using the minimum sequencing depth of the dataset as sample size (i.e. 56,651 sequences) and the dissimilarity matrix was calculated at each of the 100 iterations of this process. All these iterations were then averaged (mean) to obtain an average dissimilarity matrix. The dissimilarity indices were used to perform ordinations by non-metric multi-dimensional scaling (NMDS) with the *metaMDS* function, as well as to calculate the cumulative sum of dissimilarities during sequential propagation for each modality according to the formula:

$$\sum _{n=2}^{n=10}{BC}_{{C}_{n}{C}_{n-1}}$$

where $n$ denotes the step index and ${BC}_{{C}_{n}{C}_{n-1}}$the Bray-Curtis dissimilarity between a community ${C}_{n}$ and its predecessor community ${C}_{n-1}$.

### Identification of bacterial isolates and analysis

Identification of multiple isolates (approximately 50 per condition) consisted of colony picking using sterile pipette cones, which were then deposited into the wells of a PCR microplate containing 50 µL of PCR mix. The PCR mix consisted of 25 µL of TaKaRa mix (EmeraldAmp GT PCR Master Mix containing buffer, polymerase, dNTPs and loading buffer), 22 µL of sterile nuclease-free water, 1.5 µL of 10 µM forward primer (W02: 5’-GNTACCTTGTTACGACTT-3’) and 1.5 µL of 10 µM reverse primer (W18: 5’- GAGTTTGATCMTGGCTCAG-3’) [36]. The microplate was then placed in a thermal cycler for amplification according to the following program: initial denaturation at 94 °C for 3 min, followed by 30 cycles of denaturation (94 °C, 30 s), hybridization (50 °C, 30 s) and elongation (72 °C, 90 s). Final elongation was done at 72 °C for 10 min. Sanger sequencing of the amplicons and quality check were performed by LGC Genomics GmbH (Berlin, Germany) following the Premium Run procedure. Sequences were then imported into R, and a custom script allowed local taxonomic affiliation by BLAST against the NCBI database version 01/2023 [37] using rBLAST version 0.99.2 [38] and taxonomizr version 0.10.2 [39] packages. Two sequences presented multiple taxonomic affiliations and were discarded from the analysis.

### Statistical analysis

Statistical tests were performed using R version 4.1.2 [32]. Correlation tests were carried out using Spearman’s rank correlation test since data did not follow a normal distribution. Comparisons of means were performed after testing for normality of the residuals, and homoscedasticity using Bartlett’s test. When both conditions were met, a standard two-sided two samples *t-*test was performed. Wilcoxon rank sum test with continuity correction was used for non-normal data while Welch’s two-sided two-sample *t*-test was used for heteroscedastic data. Statistical significance of differences between proportions was assessed by using Fisher’s exact test with Benjamini-Hochberg correction for multiple comparisons.

## Results

### Effect of monophase and invert emulsion culture systems on bacterial community amplification

**Fig. 1:**
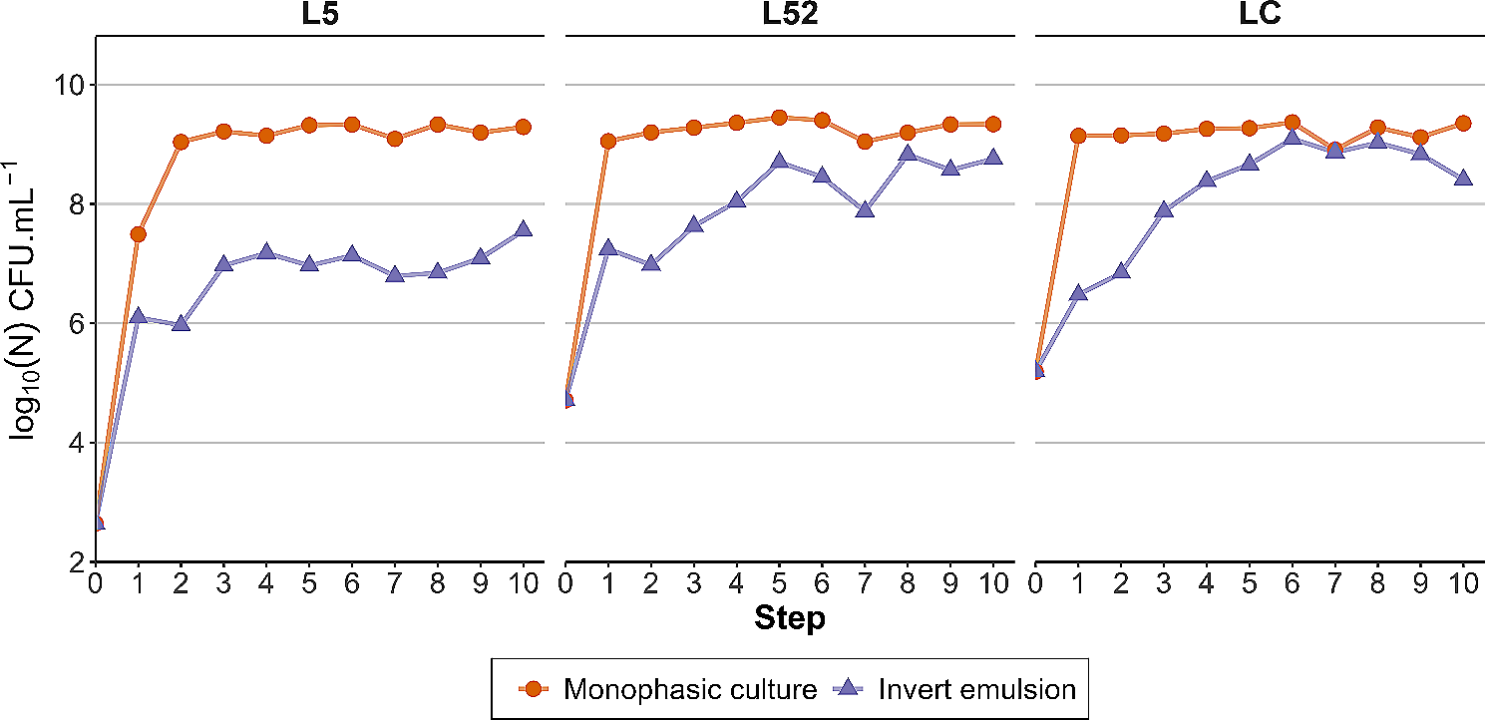
Growth dynamics of bacterial communities during serial propagation using a monophasic or an invert emulsion system

Inocula and serially propagated communities were enumerated after each step in order to investigate the ability of each culture system to amplify bacterial communities (Fig. 1). Using a monophasic setup, population densities of the L52 and LC microbial communities increased during the first growth cycle but did not change significantly afterwards (*P* ≥ 0.05, Spearman’s rank correlation from step 1 to 10), reaching a mean density of 9.27 ± 0.04 log_10_ CFU.mL^− 1^ and 9.20 ± 0.04 log_10_ CFU.mL^− 1^, respectively. The L5 community showed a similar trend with a density increase, albeit stabilizing after the second growth cycle (*P* ≥ 0.05, Spearman’s rank correlation from step 2 to 10) at 9.22 ± 0.04 log_10_ CFU.mL^− 1^ on average. These results show that the communities can reach the carrying capacity after few (1 to 2) growth cycles when propagation is performed using the monophasic setup.

Using the invert emulsion system, the density of the L5 community reached a plateau after the third propagation cycle (*P* ≥ 0.05, Spearman’s rank correlation from step 3 to 10). Density of the L52 community increased until step 5 (correlation coefficient *r*_*s*_ = 0.94 and *P* < 0.05, Spearman’s rank correlation from step 0 to 5) and remained constant thereafter (*P* ≥ 0.05). Similarly, the LC population density grew progressively until the sixth step (*r*_*s*_ = 1 and *P* < 0.001, Spearman’s rank correlation, step 0 to 6) and then stabilized (*P* ≥ 0.05).

The carrying capacity of the invert emulsion culture system was lower than the monophasic setup. Compared to the monophasic setup which achieved a final population density of 9.23 ± 0.02 log_10_ CFU.mL^− 1^ regardless of the initial raw milk, the invert emulsion led to a population density of 7.07 ± 0.08 log_10_ CFU.mL^− 1^ in L5 (*P* = 6.31.10^− 4^, Wilcoxon rank sum test with continuity correction), a density of 8.53 ± 0.14 log_10_ CFU.mL^− 1^ in L52 (*P* = 2.77.10^− 6^, Welch’s one-tailed two-sample *t*-test) and a density of 8.84 ± 0.12 log_10_ CFU.mL^− 1^ in LC (*P* = 1.61.10^− 3^, standard one-tailed two-sample *t*-test).

These results show that the increase in the population density of bacterial communities is more gradual and that the carrying capacity is generally lower when sequential propagation is performed using an invert emulsion rather than a monophase system. Overall, both systems enable the amplification of bacterial communities, although they impart different growth dynamics on them.

### Ecological dynamics of bacterial communities upon serial propagation using monophase or invert emulsion culture systems

**Fig. 2:**
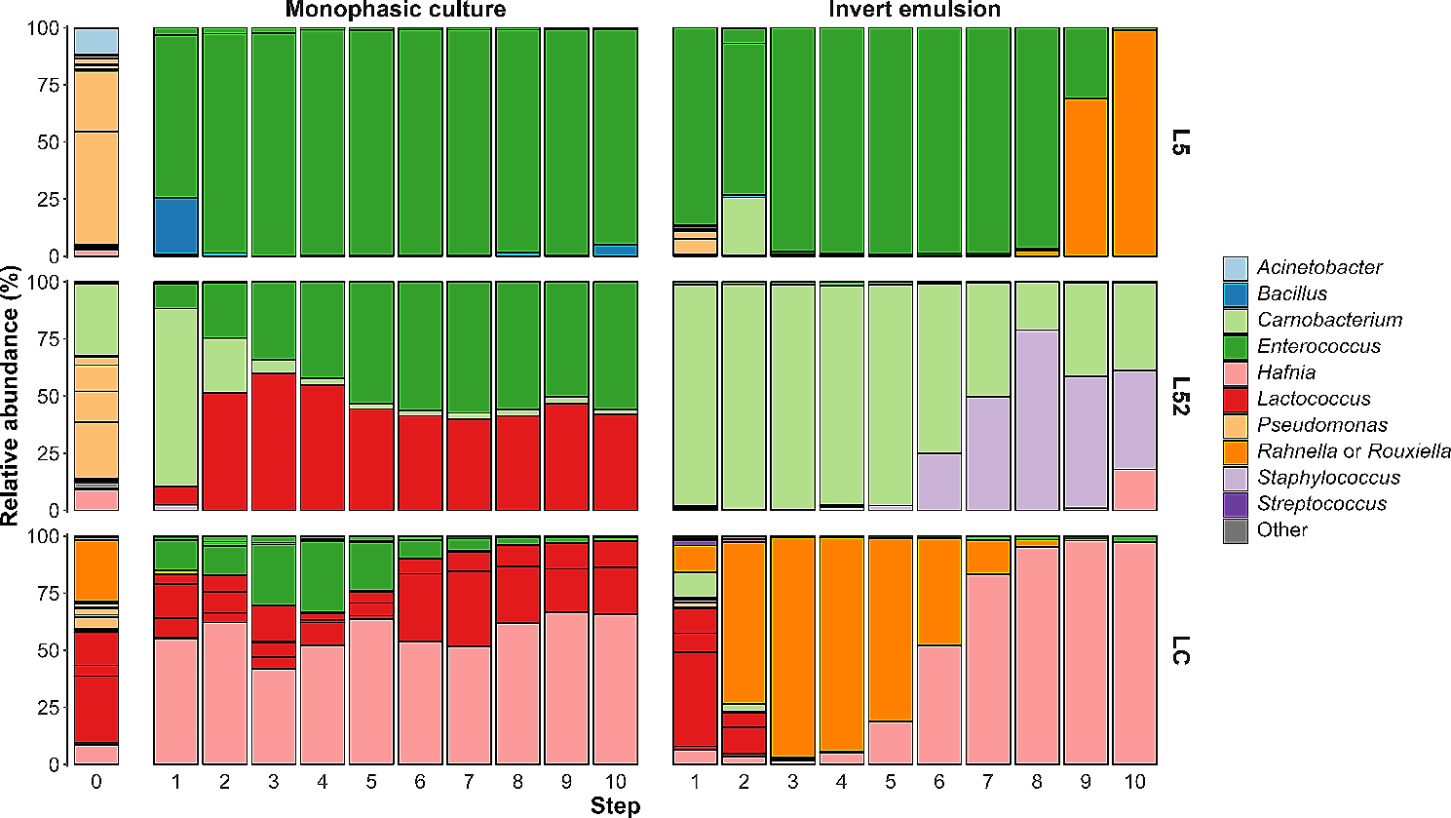
Compositional dynamics at the genus level of bacterial communities from raw milk during serial propagation using a monophasic or an invert emulsion system. Stacked bars on the left represent the inocula (noted as “0”). The ten top genera in terms of total relative abundance are shown, the others are aggregated in “Other”

In addition to characterizing community growth, inocula and communities at the end of each growth cycle (step) were analyzed by metabarcoding in order to investigate their composition during serial propagation. The compositional profiles of the propagated communities highly diverged from the originating inocula, regardless of the culture system used (Fig. 2 and Figure S1). The most drastic changes in OTU relative abundances occurred during the first propagation from step 0 to step 1. Taxa belonging to the phylum *Pseudomonadota* were largely dominant in the L5 inoculum (97.6%, step 0), before being overtaken by *Bacillota* in both monophasic system (between 99.1% and 100%, steps 1 to 10) and invert emulsion up to step 8 (between 87.4% and 99.9%), then again dominated during the last passages (68.9% at step 9 and 99.0% at step 10) (Figure S1). Similarly, the L52 inoculum was dominated by *Pseudomonadota* (67.5%, step 0), while *Bacillota* became dominant at the end of the first growth cycle, irrespective of the culture system used (monophase: 100%, invert emulsion: between 82.0% and 100%, steps 1 to 10) (Figure S1). In the LC series, *Bacillota* represented 50% of the inoculum while they represented 33.2–58.0% of the communities propagated using a monophasic setup (steps 1 to 10, Figure S1). In an invert emulsion system, *Bacillota* represented 76.4% of the community after the first growth cycle (step 1), then were supplanted by *Pseudomonadota* (steps 2 to 10) whose relative abundances reached up to 99.1% (Figure S1).

Importantly, beyond the first cycle, community compositions at the genus level tended to reach equilibrium when propagated using a monophasic culture system (Fig. 2, steps 2 to 10). Conversely, serial propagation using the invert emulsion system generated strong compositional dynamics in the communities, giving rise to wave-like patterns in the relative abundance profiles (Fig. 2, steps 1 to 10).

**Fig. 3:**
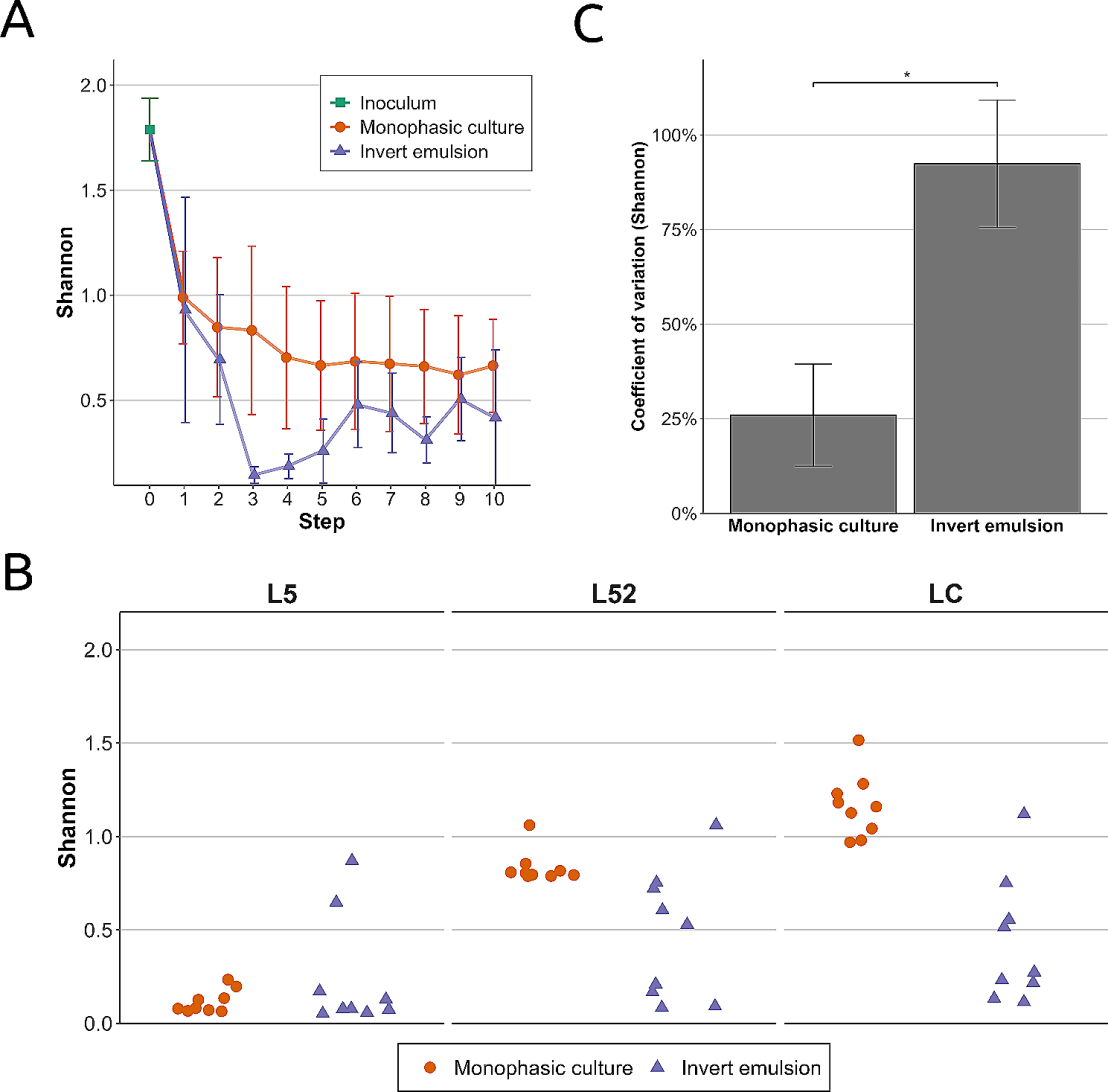
Alpha diversity analysis of raw milk communities upon serial propagation using a monophasic system or an invert emulsion. (**A**) Mean Shannon diversity index according to the growth step. (**B**) Dispersion of Shannon indices from passages 2 to 10 for each milk and culture system (**C**) Mean coefficient of variation of Shannon diversity from passages 2 to 10. Means ± standard error of the mean followed by superscript asterisks indicate levels of statistical significance based on a one-sided Welsh’s t-test. *: *P* < 0.05

Data obtained with inocula and communities propagated from the three raw milks L5, L52 and LC allowed us to investigate the effect of propagation on alpha diversity (Fig. 3). The results show that for each milk, Shannon diversity was higher in the inocula than in the propagated communities. Indeed, the mean Shannon index sharply decreased during the first growth cycle from 1.79 ± 0.14 in the inoculum (Fig. 3A, step 0, green square) to 0.99 ± 0.22 in monophasic setup (*P* = 1.49 × 10^− 2^, one-tailed paired *t*-test) and 0.93 ± 0.54 in invert emulsion, although this latter difference was not significant for this system (*P* > 0.05, one-tailed paired *t*-test). This observation is in accordance with the relative abundance profiles (Fig. 2) and denotes a gain in uniformity in propagated community structures.

However, the evolution of the Shannon index beyond the first step reveals differences between the culture systems. Indeed, the Shannon diversity of communities serially propagated using a monophasic setup showed a clear trend towards stabilization (Fig. 3A, steps 2 to 10, orange dots). Contrariwise for communities serially propagated in invert emulsion (Fig. 3A, purple triangles), the index oscillated between very low levels (0.14 ± 0.04, step 3), indicating strong dominance of a taxon in terms of relative abundance, and higher levels (0.69 ± 0.31, step 2), indicating relatively higher uniformity of OTU distribution. These results reflect the strong ecological dynamics at play during serial propagation in invert emulsion.

The wave-like patterns observed in the composition plots (Fig. 2), and reflected by the Shannon indices, emerged at different steps for the three milks. It can be observed that, whatever the starting milk, the dispersion of Shannon indices between steps was greater when communities were propagated in invert emulsion compared to the monophase system (Fig. 3B, purple triangles and orange dots, respectively). Mean coefficients of variation of Shannon indices reflect their average levels of variation for communities propagated in monophase or invert emulsion systems. The results show that the average dispersion of the Shannon index was significantly lower for communities serially propagated using a monophasic setup (26.2% ± 13.8%) than for their counterparts propagated using an invert emulsion (93.0% ± 17.2%, *P* = 1.96 × 10^− 2^) (Fig. 3C). This summarizes the trends observed previously in the succession of structures and suggests that propagation using a monophasic setup leads to community stabilization.

**Fig. 4:**
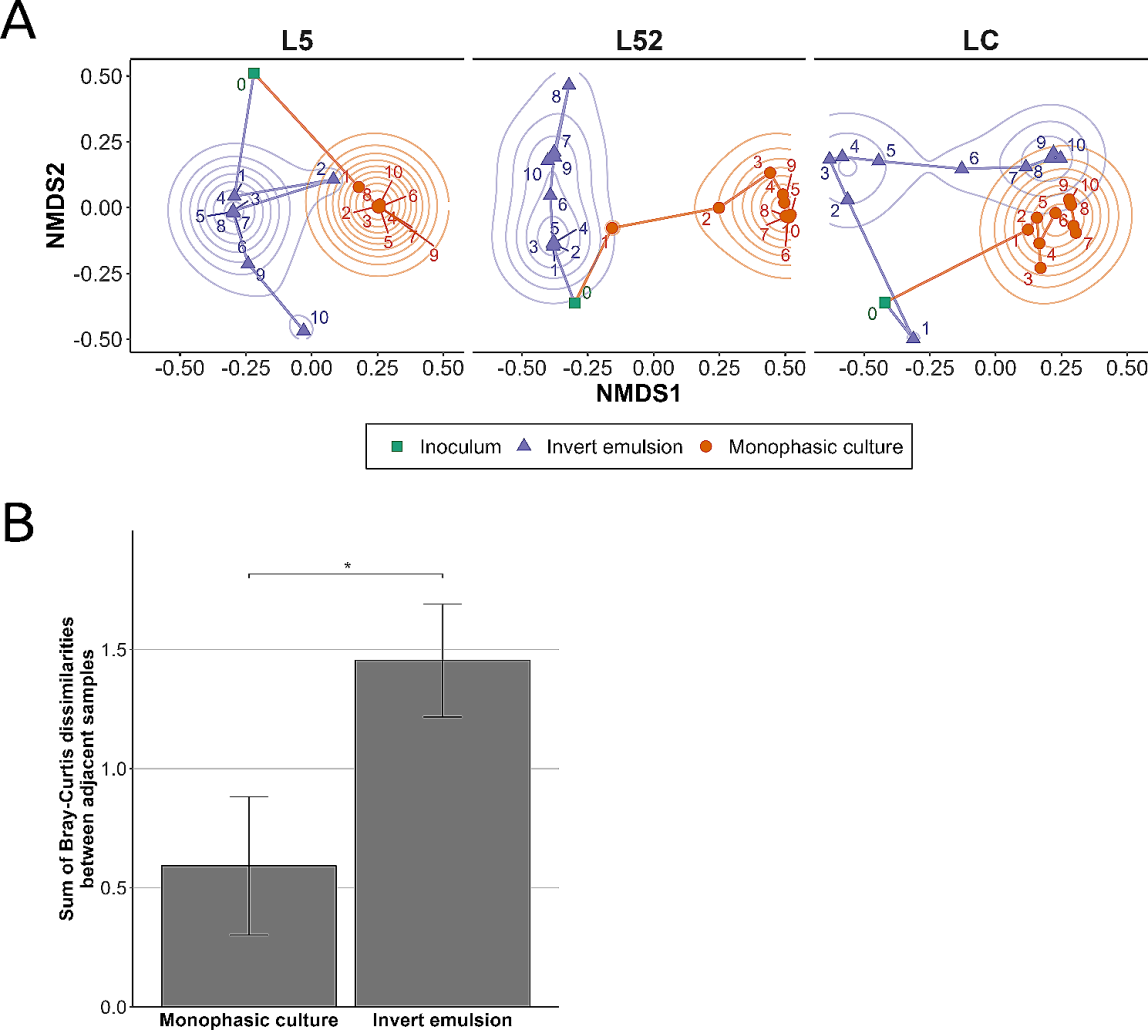
Beta diversity analysis of raw milk communities upon serial passaging using a monophasic system or an invert emulsion. (**A**) Community trajectories for each milk and culture system obtained by non-metric multi-dimensional scaling (NMDS). Concentric color ridges represent lines of equal density on the plane. **(B**) Sum of Bray-Curtis dissimilarities between adjacent communities propagated using each culture system. Means ± standard error of the mean followed by superscript asterisks indicate levels of statistical significance based on a one-tailed Welsh’s t-test. *: *P* < 0.05

A beta diversity analysis was performed for further assessment of the effects exerted by both culture systems on the structures of the serially propagated communities. Non-metric multidimensional scaling (NMDS) ordinations based on the Bray-Curtis dissimilarity matrices calculated for each series of raw milk made it possible to represent the ecological trajectories of the communities over the course of serial propagation (Fig. 4A). Regardless of the series of raw milk or the culture system considered, the long distance between the points corresponding to the propagated communities (orange dots and purple triangles) and their respective inocula (green squares) indicated a structural divergence from that of the initial community (Fig. 4A). In addition, the directions of the ecological trajectories of communities propagated using a monophase system and an invert emulsion system differ from each other (Fig. 4A, orange and purple lines). This indicates that the two culture systems orientate the structures of the propagated communities differently although they derive from the same initial raw milk community. However, the magnitude of this difference depended on the starting raw milk community. For example, after 10 propagations in monophase and emulsified systems, the points corresponding to L52-derived communities are farther apart from each other (structures are more dissimilar) than those corresponding to LC-derived communities (structures are more similar) (Fig. 4A, step 10, orange dots and purple triangles). More importantly, trajectories arising from different culture systems are also distinct in terms of their respective topologies. Overall, the points indeed corresponding to communities propagated in a monophasic setup converged towards a higher density locus as serial propagation progressed (Fig. 4A, orange dots and equidensity lines). This indicates an increase in similarity between community structures during the process and suggests that serial propagation using a monophasic setup leads to the stabilization of community structures. This observation is consistent with the stabilization of both relative abundance profiles (Fig. 2) and Shannon diversity of communities serially propagated using a monophasic setup (Fig. 3). In contrast, the points corresponding to the communities propagated in invert emulsion converged towards successive higher-density loci during serial propagation (Fig. 4A, purple triangles and equidensity lines). This suggests that the structures of these communities stabilize transiently before evolving into new structures during the process.

The means of the sums of Bray-Curtis dissimilarities between adjacent communities indicate the amount of cumulative change in community structures during serial propagation, allowing the effects of monophase and invert emulsion culture systems to be compared (Fig. 4B). The results show that on average, the cumulative sum of Bray-Curtis dissimilarities was significantly lower for the monophasic setup than for the invert emulsion system (*P* = 4.26 × 10^− 2^). This shows that on average the quantity of change in community structures was significantly lower when communities were serially propagated using a monophasic setup.

Taken as a whole, the results show that monophase and invert emulsion systems differ in the type of ecological trajectories they induce on serially propagated communities. In particular, the monophasic setup leads to early stabilization whereas the invert emulsion system leads to more extensive remodeling of serially propagated community structures.

### Enrichment of rare taxa during serial propagation in invert emulsion

**Fig. 5:**
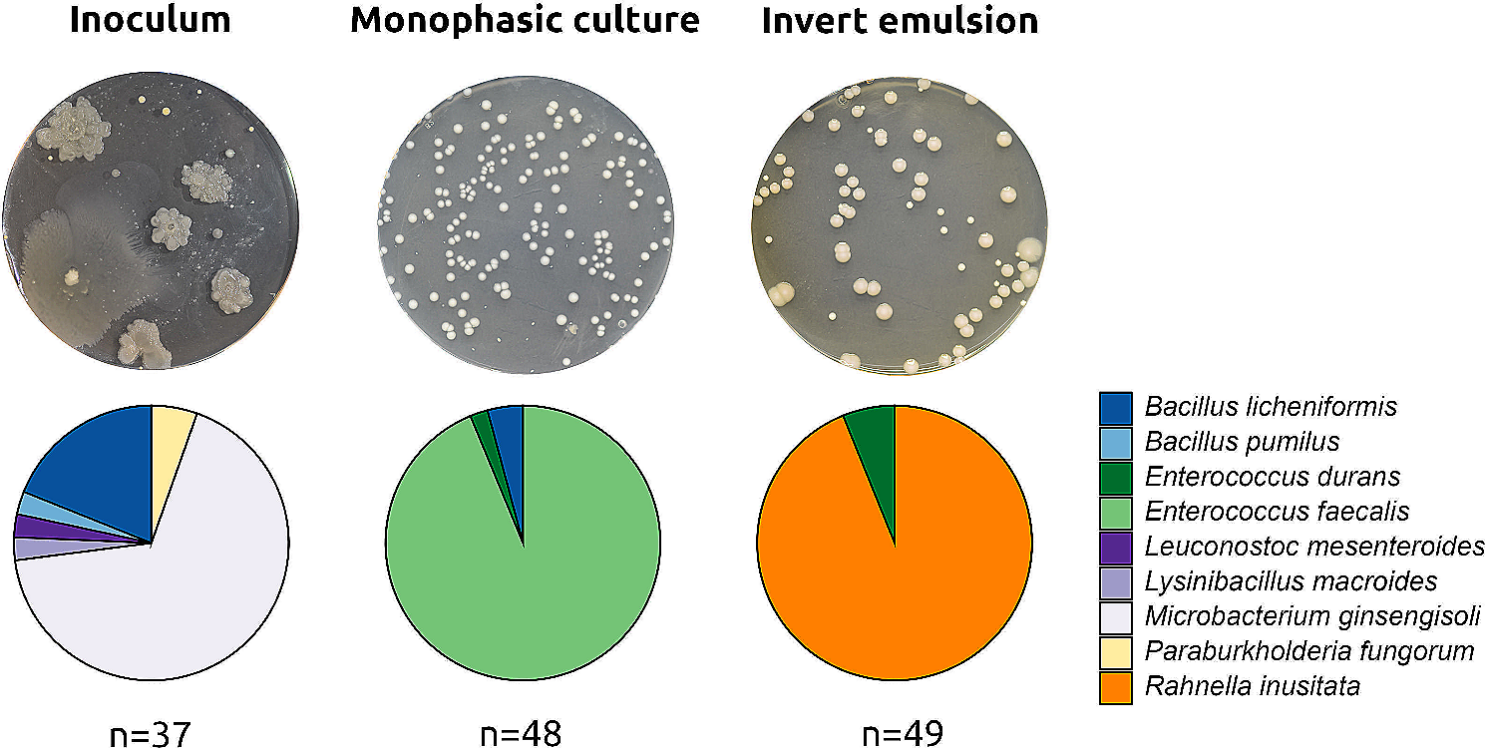
Bacterial composition of plated raw milk community L5 inoculum, and after 10 passages using a monophasic or an invert emulsion system. Top: photographs of TSAYE agar plates. Bottom: bacterial composition and corresponding taxonomic affiliation. “n” indicates the number of isolates successfully identified in each condition and without ambiguous taxonomic affiliation

Metabarcoding analysis of the bacterial communities propagated from the L5 raw milk revealed that the communities grown using the monophasic setup and the invert emulsion system have a radically different composition from each other after 10 steps (Fig. 2), which was confirmed by further analysis (Figs. 3 and 4). Interestingly, the dominant taxa in these cultivated communities, *Enterococcus* sp. and *Rahnella* or *Rouxiella* sp., were rare in the inoculum, in which they represented approximately 0.46% and 0.12% of the community respectively, whereas they represented up to 95% and 99% of the cultivated communities. These results open up the possibility of increasing the frequency of isolation of bacteria from cultured communities, compared with the lower frequency of isolation from source communities. To test this hypothesis, samples corresponding to the inoculum and the 10th steps of monophase and invert emulsion cultures were plated on TSAYE agar and incubated at 30 °C for 72 h to allow the development of colonies (Fig. 5, top). Colonies showed different morphologies under the three conditions, suggesting different taxonomies. Approximately 50 randomly selected colonies per modality were then identified by Sanger sequencing of amplicons of the 16 S rRNA gene (Fig. 5, bottom).

In the communities resulting from serial passaging in monophasic setup and in invert emulsion, the majority of the colonies analyzed corresponded to *Enterococcus faecalis* and *Rahnella inusitata*, respectively, whereas these taxa could not be isolated from the plated inoculum. This result is consistent with metabarcoding analyses of propagated communities. Moreover, no bacteria belonging to the species *E. faecalis* were isolated from the serial propagations performed with the invert emulsion system. Similarly, no bacteria from the species *R. inusitata* were isolated from the community obtained by serial propagation using a monophasic setup. Considering *R. inusitata* as a taxon of interest while grouping all others together, a Fisher’s exact test indicated a *P* < 0.001 when comparing isolate counts from the community cultivated in invert emulsion with the inoculum. Similarly, comparison with the community cultivated in monophasic setup yielded a *P* < 0.001, thereby statistically confirming the compositional differences observed between the three modalities. This result shows the value of using serial passaging in invert emulsion in addition to classical methods in order to enrich and isolate rare bacteria from an ecosystem of interest.

## Discussion

The present work aimed at evaluating the contribution of monophase and invert emulsion techniques applied to the serial propagation of microbiota from raw milk for community shaping in a top-down engineering approach. To do so, three different raw milk communities were serially propagated and their composition was characterized by metabarcoding analysis after each growth cycle. None of the systems allowed to reproduce the initial community structure, which could be explained by the loss of species not adapted to culture conditions during the first growth cycle. For example, a depletion of *Pseudomonas* spp. during the first growth cycle was observed in all cases. Similarly, *Brevibacterium linens* was unable to grow in invert emulsion system and this failure was attributed to its aerobic metabolism [19]. *Pseudomonas* spp. and *Brevibacterium linens*, as strict aerobic bacteria [40], could be unable to grow in an invert emulsion due to the low oxygen availability resulting from the oil as the continuous phase, which would act as a barrier to oxygen transfer. In addition, most *Pseudomonas* spp. do not tolerate acidic conditions [40], which may explain their decline in the monophasic setup where this genus has been replaced by *Lactococcus* spp. or *Enterococcus* spp., known acidifiers. An alternative mechanism that could have contributed to the depletion of *Pseudomonas* spp. is their possible transition to a viable but non-culturable (VBNC) state [41]. More generally, and despite the contribution of technologies such as culturomics, only a fraction of the microorganisms present in an environment are able to grow under laboratory conditions [42]. The isolation and cultivation of viable organisms from a given environmental sample in the laboratory is commonly hampered by multiple factors, such as complex nutritional requirements, dormancy or intricate cross-feeding relationships between members of the community [43].

Serial passaging in a monophasic setup led to the stabilization of communities. Firstly, enumeration showed that serial propagation led to the increase and stabilization of total bacterial density. Communities grew proficiently to reach approximately 10^9^ CFU.mL^− 1^ during the first growth cycle. The stochastic sampling effect exerted by a 100-fold dilution was likely to be weak, limiting bottlenecking which is a function of population size [10, 44]. Secondly, in the monophasic setup, a stabilization of the taxonomical structures of the communities was observed with variable levels of diversity according to the starting community. Despite different compositions of the inocula, each raw milk community structure converged towards a structure attractor as shown in ordinations of Bray-Curtis dissimilarity matrices. These equilibria were characterized by lower levels of diversity compared to the inocula. The exclusion of taxa mainly occurred during the first and second growth cycles and can be explained by two hypotheses. Firstly, a significant proportion of the original community was probably unable to grow under the laboratory culture conditions used. Secondly, bacterial interactions in the mixed culture could have led to the selection of a subset of taxa that could co-exist, and the exclusion of taxa from that subset [45]. During serial propagation, the Shannon index showed a stable trend, indicating a stabilization of the taxonomic distribution and the community structure. These results are consistent with previous reports, where serial passaging in monophasic setup is commonly associated with the stabilization of the structures of microbial communities. Taxonomical stabilization upon serial passaging was described in diverse microbiomes including those of the gut [20], plants [21, 22], activated sludge [23] and milk fermentation reactors [46]. In serial passaging setups, communities undergo ecological succession during each growth cycle but eventually reach a state of “generational equilibrium” as structures converge over successive incubations [10, 44, 47]. This compositional robustness is thought to be due to bacterial interactions in the mixed culture [12, 48–50]. Serial propagation techniques have also been subject to empirical practice for millenia. Indeed, undefined microbial communities of fermented foods are usually proliferated by “backslopping”, a serial propagation technique consisting of using remnants from previous fermentation processes to inoculate fresh raw material to initiate a new fermentation [51]. Our results showed that monophasic serial propagation can be used to propagate communities stably and that it could therefore be used in the selection process of communities with phenotypes of interest [10].

The communities serially propagated using an invert emulsion evolved differently from those serially propagated using a monophasic setup. Indeed, NMDS ordination showed that the trajectories of the communities propagated in a monophase system or in invert emulsion were divergent, despite originating from the same initial community. By shaping the structure of serially propagated communities in a different way than a monophase system, the invert emulsion could constitute a relevant tool for microbiome engineering. The succession of structures observed during serial propagation in invert emulsion suggests that this tool may be useful for investigating the effect of relative abundances of microorganisms on the functionality of cultivated communities.

NMDS ordination also revealed the presence of multiple attractors, suggesting transient stabilizations of community structure during serial propagation in invert emulsion. On the contrary, the ecological trajectories of communities propagated in a monophase system presented a single attractor, indicating the early convergence and stabilization of community structures. Thus, propagation using a monophase system is more conservative regarding the structure of serially propagated communities, once the first propagation stage has been completed. Therefore, it might be interesting to combine the two approaches, for example by first using the invert emulsion system to shape microbial communities and then the monophase system to stabilize their structures. Furthermore, the transient stability of the community structures during serial propagation in invert emulsion was also apparent on the OTU relative abundance profiles, which displayed wave-like patterns. This was reflected by oscillations of the Shannon diversity index over the entire process. It has been previously reported that bacterial segregation in invert emulsion prevents the establishment of biotic interactions [17, 19]. In such a system, fast-growing bacteria are expected to quickly dominate the distribution while slower-growing taxa are expected to appear in later steps. Hence, these emerging patterns are likely to be driven by the growth dynamics of individual bacteria entrapped into separate nurturing droplets. Consequently, the variation of community structures during serial propagation in invert emulsion is expected to result from these dynamics. These expectations are consistent with previous works reporting nutrient privatization through spatial segregation as the underlying mechanism of enrichment for slower-growing mutants during serial propagation using invert emulsions [17] or double-emulsions [52].

The succession of community structures observed in invert emulsion system suggested that it could be used for the enrichment of taxa poorly represented in the inoculum. To test this hypothesis, approximately 50 colonies were isolated from the L5 community inoculum after 10 growth cycles in monophasic setup and invert emulsion. Cultivation in an inverted emulsified medium allowed for the successful isolation of bacteria from the species *Rahnella inusitata*, which was poorly abundant in the inoculum and became dominant after 10 steps. This species was previously found to spoil milk during storage [53] but presents interesting technological properties as biocatalyst [54]. Therefore, the ability to isolate this species may be important for the investigation of milk spoilage mechanisms as well as applications in pharmaceutical engineering. The difficulty in isolating and propagating a wide variety of microorganisms from complex mixtures has long been described in the literature [55]. *“Recovering a single organism within the mixed community is akin to finding a needle in a haystack”*, especially if these microorganisms grow slowly and/or are subdominant in the population [43]. Different strategies have been proposed for the recovery of such microorganisms, such as the use of bacteriophages to eliminate targeted abundant populations [56], microfluidics for high-throughput streaking of microcultures on agar plates [57] or the high-throughput diversification of culture conditions, i.e. culturomics [58]. More recent work has enabled the enrichment and isolation of slower-growing mutants in defined and undefined communities, using a microfluidic device to encapsulate single bacteria in double emulsions and separate them during culture [52]. Our results highlight the interest of adding the invert emulsion technique to the palette of tools available for the characterization of microbiota, and isolation of rare taxa.

A high discrepancy was observed between the metabarcoding data and the taxonomic identity of the isolated bacteria for the raw milk sample. This is in contrast with the cultivated communities, for which a high congruence was found with the culture-dependent taxonomic method. For example, bacteria of the genera *Microbacterium*, *Paraburkholderia*, *Lysinibacillus* and *Leuconostoc* were isolated from the L5 raw milk sample, even though no reads matched these genera in the metabarcoding analysis. This is likely because only a small fraction of the bacterial diversity present in raw milk is able to grow in the laboratory medium used in our study. This hypothesis agrees with the diversity metrics which have shown a high decrease in diversity between the inoculum and the cultivated communities. This result emphasizes the interest in coupling culture-dependent and culture-independent approaches for the characterization of microbiotas as they both bring partial but complementary information about their composition [43]. Indeed, while DNA-based approaches such as metabarcoding can detect non-cultivable or dead microorganisms, culture-based approaches can allow the detection of low-abundance microorganisms [43].

This work has demonstrated that the invert emulsion system can be used to generate a wide range of diverse communities from a single starting consortium. Future research is needed to fully understand the potential benefits of this top-down approach to microbial community engineering. This includes fine-tuning of the emulsion parameters such as droplet size, stability and compatibility with different microbial strains to ensure efficient propagation [19]. Multi-omics approaches may help to understand how invert emulsion culture affects microbial physiology [8]. This technology could also benefit from predictive modeling to anticipate outcomes in terms of community structure and functionality [59]. Finally, this culture system could also be applied to microbial communities from other environments, such as soil, stool, aerial parts of plants and their roots, or wastewater.

## Conclusions

Intense research activities on microbiomes have led to a strong need for microbial community propagation techniques. In this work, two co-culture systems were used for the serial propagation of bacterial communities from three raw milks and the evolution of their structures was studied. An invert emulsion system, relying on the privatization of public goods through bacterial confinement in droplets was compared to a monophasic setup wherein bacteria share their resources and can establish interactions. The results showed that in a serial propagation process, the use of a monophasic culture system led to compositionally stable communities after a few steps, while the use of an invert emulsion system led to progressive compositional changes with only transient stability. In addition, the propagated communities differed according to the system, although they originated from a single inoculum, highlighting the complementarity of the tools for shaping microbiota. Finally, serial propagation in invert emulsion allowed the successful isolation of a low-abundant taxon, thereby demonstrating its usefulness as a tool to enrich the available bacterial repertoire.

### Electronic supplementary material

Below is the link to the electronic supplementary material.

Supplementary Material 1

## Data Availability

The datasets presented in this study can be found in the data repository DOREL (Données de Recherche Lorraines) at 10.12763/ULWTZH.
