# Supplementary material for: Serial cultures in invert emulsion and monophase systems for microbial community shaping and propagation
Source: Microb Cell Fact. 2024 Feb 14;23:50. doi: 10.1186/s12934-024-02322-3 (PMC10865683; doi:10.1186/s12934-024-02322-3)
Supplement: Supplementary file 1 — Supplementary Material 1 [file 12934_2024_2322_MOESM1_ESM.docx]

**Supplementary material**


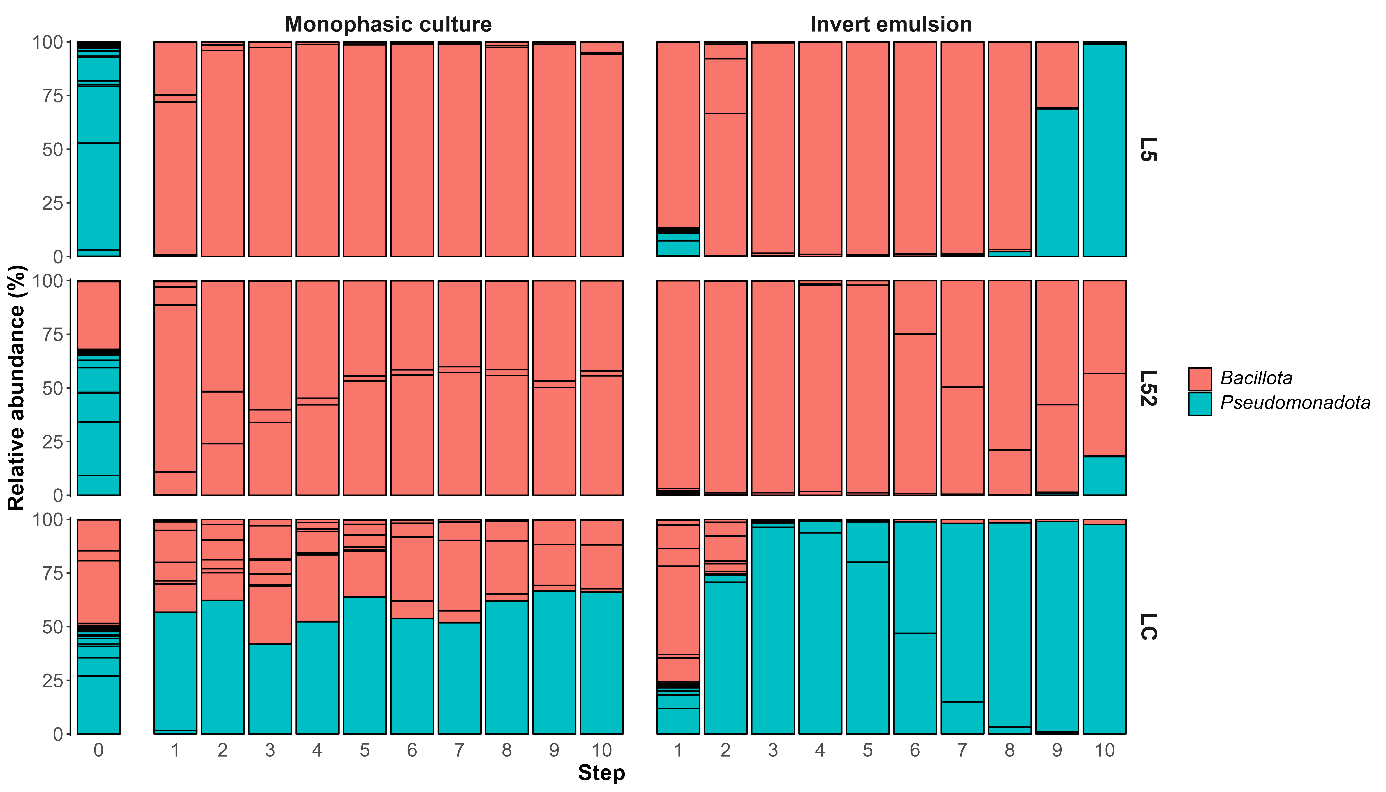


**Figure S1. Compositional dynamics at the phylum level of bacterial communities from raw milk during serial propagation using a monophasic or an invert emulsion system.** Stacked bars on the left represent the inocula (noted as “0”).
